# Supplementary material for: Methodological transparency of preoperative clinical practice guidelines for elective surgery. Systematic review
Source: PLoS One. 2023 Feb 24;18(2):e0272756. doi: 10.1371/journal.pone.0272756 (PMC9956602; doi:10.1371/journal.pone.0272756)
Supplement: S1 File — (DOCX) [file pone.0272756.s001.docx]

**Glossary of terms of included CPG**

**Preoperative fasting**

**AAA 2016:** Guidelines of the Association of Anaesthesia, Analgesia and Recovery of Buenos Aires on pre-operative fasting in adults and paediatrics in elective procedures (27)

**ASA 2011:** Practice Guidelines for Preoperative Fasting and the Use of Pharmacologic Agents to Reduce the Risk of Pulmonary Aspiration: Application to Healthy Patients Undergoing Elective Procedures. American Society of Anaesthesiologists (26)

**ASA 2017:** Practice Guidelines for Preoperative Fasting and the Use of Pharmacologic Agents to Reduce the Risk of Pulmonary Aspiration: Application to Healthy Patients Undergoing Elective Procedures. American Society of Anaesthesiologist (19)

**CAS 2019:** Preoperative fasting in patients undergoing elective surgery and procedures. College of Anaesthesiologists Singapore (28)

**ESA 2011:** Perioperative fasting in adults and children: guidelines from the European Society of Anaesthesiology (25)

**ESN 2017:** European Society for Clinical Nutrition and Metabolism. ESPEN guideline: Clinical nutrition in surgery (24)

**Cardiac assessment for non-cardiac surgery**

**AHA-ACC 2014:** American College of Cardiology/American Heart Association. Guideline on Perioperative Cardiovascular Evaluation and Management of Patients Undergoing Noncardiac Surgery (22)

**BSC 2011:** II Guidelines for Perioperative Evaluation of the Brazilian Society of Cardiology (35)

**BSC 2017:** 3rd Guideline for perioperative Cardiovascular evaluation of the Brazilian Society of Cardiology (32)

**CCS 2017:** Canadian Cardiovascular Society Guidelines on Perioperative Cardiac Risk Assessment and Management for Patients Who Undergo Noncardiac Surgery (20)

**ESA 2018:** Pre-operative evaluation of adults undergoing elective noncardiac surgery. Updated guideline from the European Society of Anaesthesiology (29)

**ESA 2011:** Preoperative evaluation of the adult patient undergoing non-cardiac surgery: guidelines from the European Society of Anaesthesiology (30)

**ESC-ESA 2014:** Guidelines on non-cardiac surgery: cardiovascular assessment and management. European Society of Cardiology (ESC) and the European Society of Anaesthesiology (21)

**GFM 2010:** Clinical practice guide for preoperative assessment in non-cardiac surgery in adults Mexico (31)

**JCS 2011:** Guidelines for Perioperative Cardiovascular Evaluation and Management for Noncardiac Surgery. Japanese Circulation Society (36)

**SAC 2016:** Argentine Consensus for the Evaluation of Cardiovascular Risk in Non-Cardiac Surgery (34)

**Use of routine preoperative tests**

**ICSI 2020:** Institute for Clinical Systems Improvement. Health Care Guideline: Perioperative (23)

**NICE 2016:** Preoperative tests (update) Routine preoperative tests for elective surgery. National Institute for Health and Care Excellence (37)

**SEA 2014:** Recommendations for preoperative tests in adult patients for procedures in outpatient surgery. Spanish Society of Anesthesiology, Resuscitation and Pain Therapeutics (38)
