# Supplementary material for: Methodological transparency of preoperative clinical practice guidelines for elective surgery. Systematic review
Source: PLoS One. 2023 Feb 24;18(2):e0272756. doi: 10.1371/journal.pone.0272756 (PMC9956602; doi:10.1371/journal.pone.0272756)
Supplement: S3 Appendix — (DOCX) [file pone.0272756.s004.docx]

**Supplementary information S3**

**Excluded CPG during eligibility process**

**Reason for exclusion: CPG for specific conditions (11)**

1. The Academy of Breastfeeding Medicine. ABM Clinical Protocol #25: Recommendations for Preprocedural Fasting for the Breastfed Infant: ‘‘NPO’’ Guidelines. Breastfeed Med 2012;7(3):197-202.Avalaible from: <https://pubmed.ncbi.nlm.nih.gov/22803929/>.
2. Thomas M, Morrison C, Newton R, Schindler E. Consensus statement on clear fluids fasting for elective pediatric general anesthesia. Paediatr Anaesth2018;28(5):411-414. Avalaible from: <https://pubmed.ncbi.nlm.nih.gov/29700894/>
3. Green SM, Leroy PL, Roback MG, Irwin MG, Andolfatto G, Babl E et al. An international multidisciplinary consensus statement on fasting before procedural sedation in adults and children. Anaesthesia 2020;75(3):374-385.Availaible from: <https://pubmed.ncbi.nlm.nih.gov/31792941/>
4. Escudero JA, Calvo JM, Veirasc S, García R, González A. Recommendations on strategy for reducing risk of heart failure patients requiring noncardiac surgery Reducing risk of heart failure patients in noncardiac surgery. Rev Esp Anestesiol Reanim 2015;62(7):359-419. Avalaible from: <https://pubmed.ncbi.nlm.nih.gov/26164471/>
5. Tew GA, Ayyash R, Durrand J, Danjoux GR. Clinical guideline and recommendations on pre-operative exercise training in patients awaiting major non-cardiac surgery. Anaesthesia 2018 ;73(6):750-768. Avalaible from: <https://pubmed.ncbi.nlm.nih.gov/29330843/>
6. Mohanty S, Rosenthal RA, Rusell MM, Neuman MD, Ko CY, Esnaola NF. Optimal Perioperative Management of the Geriatric Patient: A Best Practices Guideline from the American College of Surgeons NSQIP and the American Geriatrics Society. J Am Coll Surg 2016 ;222(5):930-47. Avalaible from: <https://pubmed.ncbi.nlm.nih.gov/27049783/>
7. Chow WB, Rosenthal RA, Merchow RP, Ko CY, Esnaola NF. Optimal Preoperative assessment of the geriatric surgical Patient: A Best Practices Guideline from the American College of Surgeons National Surgical Quality Improvement Program and the American Geriatrics Society. J Am Coll Surg 2012;215(4):453-66. Avalaible from: <https://www.journalacs.org/article/S1072-7515(12)00493-0/fulltext>
8. Bonhomme F, Ajzenberg N, Schved JF, Serge M, Samama CM.Pre-interventional haemostatic assessment Guidelines from the French Society of Anaesthesia and Intensive Care, European Journal of anaesthesiology. Eur J Anaesthesioly 2013;30(4):142-62 Avalaible from: .<https://pubmed.ncbi.nlm.nih.gov/23435255/>
9. Feely MA, Collins CS, Daniels PR, Kebede EB, Jatoi A, Mauck KF. Preoperative Testing Before Noncardiac Surgery: Guidelines and Recommendations. Am Fam Physician 2013 15;87(6):414-8.87. Avalaible from: <https://pubmed.ncbi.nlm.nih.gov/23547574/?from_term=preoperative+guidelines&from_filter=ds1.y_10&from_pos=6>
10. Edwards D, Hedrick TL, Jayaram J, Argoff C, Gulur P, Holubar SD et al. American Society for Enhanced Recovery and Perioperative Quality Initiative Joint Consensus Statement on Perioperative Management of Patients on Preoperative Opioid Therapy. Anesth Analg 2019 Aug;129(2):553-566.Avalaible from: <https://pubmed.ncbi.nlm.nih.gov/30768461/>
11. Chambrier C, Sztark F, Société Francophone de nutrition clinique et métabolisme (SFNEP), the Société franc¸aise d’anesthésie et réanimation (SFAR). French clinical guidelines on perioperative nutrition. Update of the 1994 consensus conference on perioperative artificial nutrition for elective surgery in adults. J Visc Surg 2012 Oct;149(5):e325-36.Avalaible from: <https://pubmed.ncbi.nlm.nih.gov/23107793/>

**Reason for exclusion: Language of publication (3)**

1. Deutsche Gesellschaft für Anästhesiologie und Intensivmedizin (DGAI), Deutsche Gesellschaft für Innere Medizin (DGIM), Deutsche Gesellschaft für Chirurgie (DGCH). Präoperative Evaluation erwachsener patienten vor elektiven, nicht Herz-Thorax-chirurgischen Eingriffen. Anaesthesist 2017 -66:442–458. Available from: <https://link.springer.com/article/10.1007/s00101-017-0321-5>
2. Gemeinsame Empfehlung der Deutschen Gesellschaft für Anästhesiologie und Intensivmedizin, der Deutschen Gesellschaft für Chirurgie und der Deutschen Gesellschaft für Innere Medizin. Präoperative evaluation erwachsener patienten vor elektiven, nichtkardiochirurgischen Eingriffen. Kardiologe 2011- 5:13–26. Avalaible from: <https://leitlinien.dgk.org/files/2011_Empfehlungen_Praeoperative_Evaluation.pdf>
3. Société française d’anesthésie et de réanimation (Sfar), Société française de cardiologie (SFC). Perioperative assessmentof cardiac risk patient in non-cardiac surgery. Annales Françaises d’Anesthésie et de Réanimation 30-2011 e5–e29.Avalaible from:<https://sfar.org/prise-en-charge-du-coronarien-opere-en-chirurgie-non-cardiaque/>
