# Supplementary material for: Methodological transparency of preoperative clinical practice guidelines for elective surgery. Systematic review
Source: PLoS One. 2023 Feb 24;18(2):e0272756. doi: 10.1371/journal.pone.0272756 (PMC9956602; doi:10.1371/journal.pone.0272756)
Supplement: Attachment — Submitted filename: PLOS ONE manuscript review.docx [file pone.0272756.s005.docx]

**Title:** methodological transparency of preoperative clinical practice guidelines for elective surgery. Systematic review.

This is a timely and very important study. Congratulations to all authors.

Please note the following suggestions to further improve the quality of the manuscript:

Line 29: need to be corrected as “..increase quality while decreasing the risk of…”

Line 138: need to be corrected as “All evaluators underwent a thorough detailed training…”

Line 272: need to be corrected as “…interest were declared..”

Line 273: however, it is better to report the proportion of CPG that reported the funding source here.

Line 276: score of 67% cannot be considered as a ‘high score’

Line 276: need to be corrected as “…(67%), with higher scores for CPG..”
