# Supplementary material for: Methodological transparency of preoperative clinical practice guidelines for elective surgery. Systematic review
Source: PLoS One. 2023 Feb 24;18(2):e0272756. doi: 10.1371/journal.pone.0272756 (PMC9956602; doi:10.1371/journal.pone.0272756)
Supplement: Attachment — Submitted filename: Response to Reviewers.docx [file pone.0272756.s006.docx]

December 3 of 2022

**Professor**

**Seung-Hwa Lee**

**Academic Editor**

**PLOS ONE**

I would like to acknowledge for the special and learning-focused reviews received in our manuscript (PONE-D-22-20952) entitled “Methodological transparency of preoperative clinical practice guidelines for elective surgery. Systematic review”.

We aimed to solve all comments of the reviewers and follow all suggestions to improve the quality and transparency of our paper. We carefully considered all reviewers’ comments, and we are submitting a detailed list of responses to each comment with quotes from the text.

We hope that our manuscript can fully meet all the PLOS ONE’s publication criteria.

Thank you for your consideration.

Sincerely,

Jose A. Calvache on behalf of the authors

**Responses to each comment.**

| **General comments** |  |
| --- | --- |
| 1. Please ensure that your manuscript meets PLOS ONE's style requirements, including those for file naming. The PLOS ONE style templates can be found at  https://journals.plos.org/plosone/s/file?id=wjVg/PLOSOne_formatting_sample_main_body.pdf and  https://journals.plos.org/plosone/s/file?id=ba62/PLOSOne_formatting_sample_title_authors_affiliations.pdf | We updated all details of our manuscript to follow in detail each formatting requirement. We also adjusted details regarding titles, affiliations, sections of the manuscript, supporting information (Appendix), citations and references. |
| 2. Please amend your current ethics statement to include the full name of the ethics committee/institutional review board(s) that approved your specific study. | Done. Under our current country regulation, this type of study (a literature review) does not need an ethical approval or institutional board approval. We are not directly including patients and collecting the available information for synthesis. We adjusted the sections details by following your comment. |
| 3. Please ensure that you have specified (1) whether consent was informed and (2) what type you obtained (for instance, written or verbal, and if verbal, how it was documented and witnessed). If your study included minors, state whether you obtained consent from parents or guardians. If the need for consent was waived by the ethics committee, please include this information. | Done. Please see the comment above. We do not have any informed consent while we are collecting data of published literature. |
| 4. Thank you for stating the following in the Acknowledgments Section of your manuscript:  This work was supported by departmental funding (Department of Anesthesiology, Universidad del Cauca, Colombia, and Department of Anesthesiology, Erasmus University Medical Centre Rotterdam, The Netherlands).  We note that you have provided funding information that is not currently declared in your Funding Statement. However, funding information should not appear in the Acknowledgments section or other areas of your manuscript. We will only publish funding information present in the Funding Statement section of the online submission form.  Please remove any funding-related text from the manuscript and let us know how you would like to update your Funding Statement. Currently, your Funding Statement reads as follows:  The funders had no role in study design, data collection and analysis, decision to publish, or preparation of the manuscript.  Please include your amended statements within your cover letter; we will change the online submission form on your behalf. | Corrected. We have adjusted the section deleting the funding support from acknowledgments section and moving it to Funding Statement. We apologize for the inconvenience.  Quote from Funding Statement: “This work was supported by departmental funding (Department of Anesthesiology, Universidad del Cauca, Colombia, and Department of Anesthesiology, Erasmus University Medical Centre Rotterdam, The Netherlands). The funders had no role in study design, data collection and analysis, decision to publish, or preparation of the manuscript.”.  We included this adjustment in our cover letter. |
| 5. Please include captions for your Supporting Information files at the end of your manuscript, and update any in-text citations to match accordingly. Please see our Supporting Information guidelines for more information: http://journals.plos.org/plosone/s/supporting-information. | Done. We adjusted all our Appendix by following the Supporting Information guidelines section. We adjusted their names (to Appendix S1, etc.).  We added at the end of the manuscript captions about Supporting information presented in this manuscript |
| 6. Please review your reference list to ensure that it is complete and correct. If you have cited papers that have been retracted, please include the rationale for doing so in the manuscript text, or remove these references and replace them with relevant current references. Any changes to the reference list should be mentioned in the rebuttal letter that accompanies your revised manuscript. If you need to cite a retracted article, indicate the article’s retracted status in the References list and also include a citation and full reference for the retraction notice. | Done. We checked in detail each reference to adjust them and ensure they are correct. We |

| **Reviewer #1** |  |
| --- | --- |
| This is a timely and very important study. Congratulations to all authors. | Thank you for the comment. |
| Line 29: need to be corrected as “..increase quality while decreasing the risk of…” | Corrected. Thank you for the grammar improvements in our manuscript. |
| Line 138: need to be corrected as “All evaluators underwent a thorough detailed training…” | Corrected. |
| Line 272: need to be corrected as “…interest were declared..” | Corrected |
| Line 273: however, it is better to report the proportion of CPG that reported the funding source here. | Done. We agree and we added a last line to this paragraph. Quote: “All CPG classified as recommended reported their funding sources while none of the recommended with modifications did.”. |
| Line 276: score of 67% cannot be considered as a ‘high score’ | Corrected. We adjusted the paragraph as follows. Quote: “…included guidelines presented a median score of 67%, with higher scores for CPG produced in…”. |
| Line 276: need to be corrected as “…(67%), with higher scores for CPG..” | Corrected. Please see the previous comment. |

| **Reviewer #2** |  |
| --- | --- |
| - In the “search strategy” item, the authors cite the "TRIP Database", but in the "Data sources" item of the abstract it is not mentioned; | Corrected. We adjusted the abstract section to reflect the same information that the methods section. Quote: “…in Pubmed MEDLINE, TRIP Database, Embase, the Cochrane Library…”.  We also added to Appendix 2 detailed information about the search strategy used in TRIP Database. |
| - The authors don´t mention when the search in databases was conducted; | Corrected. We added he relevant information regarding dates of our searches at the end of the paragraph. Quote “All searches were performed in December 2021 and updated in June 2022.”. |
| - Could you provide list of excluded texts in the supplement (second elegibility)? Could you provide table with the characteristics of the guidelines in the supplement? | Done. We described some details of the excluded CPG (14) from our full-text assessment in the Supporting information S3 (S3 Appendix). We create this new Supporting information File to clarify our 14 excluded texts (second eligibility).  We added to the first line of the results section. Quote: “We included 20 CPG in our analysis (Figure 1). During our eligibility process, we excluded 14 CPG, text in S3 Appendix”. |
| - Line 185: "...only six (20%) reported a formal method for achieving..." – the % should be 30% (6 of 20); | Corrected. Thanks for checking in detail this proportion. We apologize for this typo. Corrected by 30%. |
| -Table 1, in the part where it presents data on clear reporting of funding reports only from 10 guidelines, could it not include the option of "not mentioned" or "not clearly mentioned"? | Done. Indeed, we describe only the option for the positive answer to “clearly reported”. To clarify, and following the comment, we added a second line with the option “not mentioned”, which is complementary as a binary variable. |
| - In the discussion (lines 245/246): "...obtained AGREE-II scores above 90%;", I believe it should be equal or above 90%, since one of the guidelines presented a score of 90%; | Corrected. We agree. We adjusted the text to “…obtained AGREE-II scores equal or above 90%;”. |
| - Domain 6 had a median of 97%, but there were guidelines with low scores (0) in this domain, and it bothered me not to comment on this variability; | Done. To discuss regarding this topic, we added a final paragraph to the discussion section. We would like to emphasize the importance of editorial independence during the process of development of a CPG.  Quote: “Across domains, editorial independence obtained the highest score (97%) but there are some CPG scoring zero in this domain. While this finding adds confidence to the provided recommendations by reducing the risk of delivering biased recommendations or being influenced by sponsors (seeking their benefits) or by the pharmaceutical industry, there are still some CPG produced without a clear and transparent editorial independence. This large scoring variability also reflects the relative importance each sponsor gives to the editorial independence process during development, and the great implications to reviewers and evaluators during judging this domain”. |
| - Couldn't a "limitation" be to have considered versions of the same guideline? And language? | Done and completed. We added a final paragraph to the section of limitation to clarify regarding these limitations.  Quote: “Third, to include updated versions of certain CPG may be controversial but it also allows us to compare the change in score with the previous versions. Finally, while most CPG are published in English, we included only CPG published in English and Spanish losing some relevant information from particular countries.”. |
| - In the results topic, I was bothered by the fact that he reported that 14 guidelines were classified as recommended and mentioned table 1, which despite presenting separate data according to quality classification, is more focused on characteristics, while the evaluation result/scores are in table 2; | We agree with the reviewer.  The classification was the result of the data presented in the Table 2 but considering the Table 1 stratify the information between recommended vs recommended with modifications, we added as a source of the classification both tables. So, the reader can check how the CPG were classified as well as their characteristics as the first approach to their judgement. We hope this add solve this concern.  Quote: “…to be implemented in clinical practice (Tables 1 and 2).”. |
| - It is missing an arrow in the flowchart (Figure 1); | Corrected. We added the missing arrow. |
